# Immunological and clinical overlap between autoimmune gastritis and autoimmune liver diseases: a prospective cohort study

**DOI:** 10.3389/fimmu.2025.1628478

**Published:** 2025-07-25

**Authors:** Sara Massironi, Giulia Dispinzieri, Alberto Rossi, Laura Cristoferi, Marco Vincenzo Lenti, Alessio Gerussi, Alessandra Elvevi, Marco Carbone, Alessandra Bonfichi, Antonio Di Sabatino, Silvio Danese, Pietro Invernizzi

**Affiliations:** ^1^ Vita e Salute San Raffaele University, Medicine and Surgery, Milan, Italy; ^2^ Department of Medicine and Surgery, University of Milano-Bicocca, Monza, Italy; ^3^ Department of Gastroenterology, Azienda Socio Sanitaria Territoriale (ASST) Grande Ospedale Metropolitano Niguarda, Milano, Italy; ^4^ Division of Gastroenterology, Center for Autoimmune Liver Diseases, European Reference Network of Hepatological Diseases (ERN RARE-LIVER), IRCCS Fondazione San Gerardo dei Tintori, Monza, Italy; ^5^ Department of Internal Medicine and Medical Therapeutics, University of Pavia, Pavia, Italy; ^6^ First Department of Internal Medicine, Fondazione Istituto di Ricovero e Cura a Carattere Scientifico (IRCCS) Policlinico San Matteo, Pavia, Italy; ^7^ Gastroenterology and Endoscopy, Istituto di Ricovero e Cura a Carattere Scientifico (IRCCS) San Raffaele Hospital, Milan, Italy

**Keywords:** autoimmune gastritis, autoimmune liver disease, primary biliary cholangitis, anti-parietal cell antibodies, intestinal metaplasia, gastric autoimmunity

## Abstract

**Background:**

Autoimmune gastritis (AIG) and autoimmune liver diseases (AILDs)—including autoimmune hepatitis (AIH), primary biliary cholangitis (PBC), and primary sclerosing cholangitis (PSC)—are chronic organ-specific immune-mediated disorders. While both conditions frequently co-occur with other autoimmune diseases, the prevalence, clinical overlap, and immunological associations between AIG and AILDs remain underexplored.

**Objective:**

To investigate the prevalence of AIG in patients with AILD and characterize the clinical, serological, and histopathological features of this overlap, to improve early detection and guide integrated management strategies.

**Methods:**

We conducted a prospective study on 104 patients with a confirmed diagnosis of AILD. All participants were screened for anti-parietal cell antibodies (APCA); those testing positive underwent upper gastrointestinal endoscopy and gastric biopsies. Histological assessment was based on the updated Sydney System, with evaluation of mucosal inflammation, glandular atrophy, and intestinal metaplasia.

**Results:**

APCA positivity was observed in 22.1% of AILD patients, with a female predominance (78.3%). The median age of AIG diagnosis in APCA-positive patients was 58 years. Among APCA-positive individuals, histological confirmation of AIG was achieved in 91.3%, with a high rate of intestinal metaplasia (95.7%) and variable OLGA stages of gastric atrophy. Comorbid autoimmune conditions were common, with 43.5% of APCA-positive patients also presenting with autoimmune thyroiditis. Notably, PBC was disproportionately represented in the APCA-positive subgroup (47.8%) compared to the overall cohort (39.0%).

**Conclusion:**

This study highlights a clinically significant association between AIG and AILDs, particularly in patients with PBC and concurrent autoimmune conditions. Given the elevated risk of gastric mucosal atrophy and potential neoplastic transformation, targeted screening for AIG in AILD patients—especially those with APCA positivity or thyroid autoimmunity—should be considered. These findings underscore the importance of cross-specialty surveillance and open new avenues for research into shared immunopathogenic mechanisms.

**Lay Summary:**

This study found that a significant number of patients with autoimmune liver diseases, especially those with primary biliary cholangitis, also show signs of autoimmune gastritis. These results support the consideration of targeted screening for gastric involvement in selected patients to improve early detection and clinical management of associated complications.

## Introduction

Autoimmune diseases constitute a heterogeneous group of conditions defined by a loss of immunological tolerance to self-antigens, resulting in targeted immune-mediated destruction of host tissues. Among these, autoimmune gastritis (AIG) and autoimmune liver diseases (AILDs)—including autoimmune hepatitis (AIH), primary biliary cholangitis (PBC), and primary sclerosing cholangitis (PSC)—represent typical forms of organ-specific autoimmunity, distinguished by chronic progressive inflammation and a substantial risk of long-term morbidity (1–4). Although each disease entity demonstrates distinct pathophysiological features, these conditions may share overlapping immunological mechanisms, genetic predispositions, and serological profiles, suggesting a potential pathophysiological link (4–7). AILD represents a major clinical challenge due to its progressive nature, often culminating in cirrhosis, liver failure, or the need for liver transplantation (8). These conditions frequently coexist with other autoimmune disorders, reflecting the systemic nature of immune dysregulation in affected patients (9).

AIG is a chronic inflammatory condition characterized by immune-mediated destruction of the gastric parietal cells, leading to atrophy of the oxyntic mucosa, decreased acid secretion, and ultimately to malabsorption and vitamin B12 deficiency (10–14). It is frequently associated with other autoimmune conditions, particularly autoimmune thyroiditis, type 1 diabetes, and vitiligo—forming part of the autoimmune polyendocrine syndrome (APS) spectrum (15). Moreover, AIG can result in several complications, including intestinal metaplasia, gastric atrophy, and neoplastic conditions (16). Even if the risk of developing gastric cancer remains debatable (17, 18), it is recognized as a condition of possible long-term neoplastic complications such as neuroendocrine neoplasms (gNENs) (19). Due to these risks, AIG is a condition that requires individualized endoscopic surveillance to monitor the progression of atrophy and the development of neoplastic changes (20–22).

Although the link between AILD and AIG, is not completely clarified and the prevalence of AIG in AILD patients has not been extensively studied, the literature suggests significant clinical overlap (10, 23–25). Shared features include similar autoantibody profiles and HLA-associated genetic susceptibility factors (26), as demonstrated by insights from genome-wide association studies (27). Furthermore, the frequent coexistence of autoimmune conditions, such as thyroid disease, Sjögren’s syndrome, and type 1 diabetes, for both AILD and AIG, highlights the systemic nature of these disorders (28, 29). This underscores the systemic nature of autoimmunity and the need for an integrated diagnostic and therapeutic approach.

This study aims to address this gap by investigating the prevalence and characteristics of AIG in a cohort of patients with AIH, PSC, and PBC, assessing whether patients with AILD are at increased risk of developing AIG and offering novel insights that may refine clinical surveillance protocols, facilitate earlier diagnosis, and inform the development of more targeted therapeutic strategies.

## Methods

### Study design, setting, and participants

This prospective cohort study was conducted at the IRCCS San Gerardo Dei Tintori, a tertiary care Center specializing in autoimmune diseases. All consecutive adult patients with an existing or new diagnosis of AILD, including AIH, PBC, and PSC, between January 2019 and December 2022 were considered for inclusion, provided they gave informed consent and had complete data for AIG evaluation. As part of the study protocol, all patients were tested for the presence of anti-parietal cell antibodies (APCA), which were measured using a standardized ELISA method. In our institution, APCA are not part of the routine autoantibody screening panel for AILD and was specifically tested as part of this prospective study protocol. Those who tested positive for APCA were proposed to undergo esophagogastroduodenoscopy (EGD) with biopsy to assess for the presence of AIG and gastric complications.

Patients were included if they gave their consent to participate in the study. The study complied with the Declaration of Helsinki and was approved by the Institutional Review Board (ID 4940).

### Data collection

Prospective data were collected through an electronic database, which compiled comprehensive clinical, laboratory, and diagnostic information. This included demographic data (age, sex, date of birth), detailed records of liver and other autoimmune diseases, and results of autoantibody panels including APCA. Patients who tested positive for APCA underwent EGD with biopsies.

### Inclusion and exclusion criteria

All adult patients (aged 18 and above) with confirmed diagnoses of AIH, PSC, or PBC based on appropriate clinical, biochemical, and histological criteria were eligible for inclusion. Patients were excluded if they did not consent to participate in the study or had incomplete data regarding the status of AIG.

### Endoscopic examination

EGD was performed in all patients who tested positive for APCA, as part of the diagnostic work-up for AIG. EGD procedures were conducted using high-definition endoscopes, operated by experienced endoscopists at IRCCS San Gerardo dei Tintori.

During the examination, the gastric mucosa was carefully inspected, with particular attention to the body and fundus regions—areas typically affected in AIG. The presence of mucosal abnormalities, including pallor, visible submucosal vessels, or nodularity, was recorded. Any evidence suggestive of atrophic changes or neoplastic lesions was photo-documented.

Standardized biopsy sampling was performed according to the updated Sydney System protocol (30, 31). A minimum of five gastric biopsies were obtained from predefined sites: two from the antrum (lesser and greater curvature, approximately 3 cm from the pylorus), two from the corpus (lesser and greater curvature, approximately 8 cm from the cardia), and one from the incisura angularis (30). Additional targeted biopsies were taken in the presence of suspicious lesions or mucosal abnormalities.

Biopsy samples were fixed in formalin, embedded in paraffin, and stained with hematoxylin-eosin. Histological evaluation focused on confirming features of chronic atrophic gastritis, assessing for the presence of intestinal metaplasia, and excluding dysplasia or neoplastic transformation. *H. pylori* status was assessed using Giemsa staining and/or immunohistochemistry as needed.

Endoscopic findings were integrated with serological and histological data to support the diagnosis of AIG. In patients with moderate to severe atrophy or intestinal metaplasia, follow-up EGD was recommended according to current international guidelines to monitor for progression and assess neoplastic risk (20).

### Histological examination

Gastric biopsies obtained during EGD from APCA-positive patients were subjected to comprehensive pathological examination using the Sydney System (30). This system standardizes the assessment of gastritis by grading and classifying the gastric mucosa based on several histological criteria: i) Inflammation: The presence and degree of inflammation were evaluated, noting the density of inflammatory cells (lymphocytes and plasma cells) in both the antral and corpus mucosa. ii) Atrophy: Atrophy was assessed by examining the loss of glandular structures in the gastric mucosa. This involves determining whether the glandular loss is focal, multifocal, or diffuse, and noting the regions (antrum, body) most affected. iii) Activity: This refers to the presence of neutrophilic activity within the glandular and surface epithelium, indicative of active inflammation. iv) *H Pylori* infection. v) Intestinal Metaplasia: The presence of intestinal metaplasia was recorded, characterized by the replacement of the native gastric epithelial cells with intestinal-type cells, including goblet cells. Each of these parameters was graded on a scale from 0 (absent) to 3 (severe), allowing for a quantitative assessment of the gastric mucosal status. The sum of these grades provided a comprehensive score, which could be used to classify the severity of gastritis. The results of the biopsies examination were integrated with clinical data and serological markers to aid in the comprehensive diagnosis of AIG in the context of other AILD.

### Outcome measures

The primary outcome was the diagnosis of AIG, confirmed through histological examination of gastric biopsies and positive EGD findings. Secondary outcomes included the detection of gastric complications associated with AIG, including intestinal metaplasia, gastric atrophy, gNENs, and gastric cancer.

### Statistical analysis

Descriptive statistics summarized the cohort characteristics. The prevalence of AIG was calculated and compared across different liver disease types using the Chi-square test.

Comparisons between patients with and without AIG were performed using chi-square tests or Fisher’s exact tests for categorical variables, and t-tests or Mann-Whitney U tests for continuous variables.

A p-value of less than 0.05 was considered statistically significant. Statistical analyses were performed using GraphPad Prism version 6.00 for Windows, GraphPad Software, California USA, www.graphpad.com.

## Results

### Overall AILD cohort

A total of 104 patients with AILD who were consecutively evaluated at IRCCS San Gerardo Dei Tintori, Monza, Italy, were enrolled in the study.

Among these patients, 76 (73.1%) were female and 28 (26.9%) were male, with a mean age of 52 years (range 18–75 years) (**Table 1**).

**Table 1:** Overall AILD cohort baseline characteristics.

Characteristics	Overall AILD cohort (n = 104)
Age (median, range)	57 years (20–88 years)
Gender, n (%)	
Female	76 (73.1%)
Male	28 (26.9%)
AILD Diagnosis, n (%)	
Autoimmune Hepatitis (AIH)	33 (31.7%)
Primary Biliary Cholangitis (PBC)	41 (39.4%)
Primary Sclerosing Cholangitis (PSC)	14 (13.5%)
AIH-PBC Overlap Syndrome	3 (2.9%)
AIH-PSC Overlap Syndrome	5 (4.8%)
Other AILD (Undefined cholangiopathy, Biliary cirrhosis, IgG4-related disease)	8 (7.7%)
Autoimmune Comorbidities, n (%)	
Presence of other autoimmune diseases	52 (50.0%)
- Sjögren’s Syndrome	14 (13.5%)
- Rheumatoid Arthritis	10 (9.6%)
- Systemic Lupus Erythematosus (SLE)	6 (5.8%)
- Other autoimmune diseases (vitiligo, diabetes, autoimmune encephalopathy, immune thrombocytopenic purpura, etc.)	22 (21.2%)
Autoantibody Profile, n (%)	
Anti-nuclear Antibodies (ANA)	60 (57.7%)
Anti-mitochondrial Antibodies (AMA)	30 (28.8%)
Anti-smooth Muscle Antibodies (ASMA)	20 (19.2%)
Anti-parietal Cell Antibodies (APCA)	23 (22.1%)

PBC was the most prevalent condition, diagnosed in 41 patients (39.4%), followed by AIH in 33 patients (31.7%), while PSC was present in 14 patients (13.5%). Three patients (2.9%) had an overlap picture of AIH-PBC. Five patients (4.8%) had an overlap syndrome of AIH-PSC.

Eight patients (7.7%) had other AILDs, further classified as: Undefined cholangiopathy in 4 patients (3.8%); Biliary cirrhosis in 1 patient (1.0%); Undefined primary/secondary sclerosing cholangitis in 1 patient (1.0%); IgG4-related disease in 2 patients (1.9%).

52 patients (50% of the cohort) had comorbid autoimmune conditions beyond their liver disease. The most frequently reported were Sjögren’s Syndrome in 14 patients, Rheumatoid Arthritis in 10 patients, and Systemic Lupus Erythematosus (SLE) in 6 patients. Other specific autoimmune conditions (such as vitiligo, diabetes, autoimmune encephalopathy, and immune thrombocytopenic purpura) were documented in 22 patients. The presence of other autoimmune markers varied, with antinuclear antibodies (ANA) found in 60 patients (57.7%), anti-mitochondrial antibodies (AMA) in 30 (28.8%), and anti-smooth muscle antibodies (ASMA) in 20 (19.2%).

All patients were tested for APCA.

### APCA-positive population

Out of the 104 patients, 23 (22.1%) tested positive for APCA (**Table 2**). The majority of these patients were female, accounting for 78.3% (18/23), while 21.7% (5/23) were male. The mean age of the cohort was 55.5 years (range: 20–77 years).

**Table 2:** Characteristics of APCA-positive patients.

Characteristics	APCA-positive group (n = 23)
**Age (median, range)**	58 years (20–77 years)
Gender, n (%)	
Female	18 (78.3%)
Male	5 (21.7%)
AILD Diagnosis, n (%)	
Autoimmune Hepatitis (AIH)	6 (26.1%)
Primary Biliary Cholangitis (PBC)	11 (47.8%)
Primary Sclerosing Cholangitis (PSC)	2 (8.7%)
AIH-PBC Overlap Syndrome	0 (0%)
Other AILD (Biliary cirrhosis)	1 (4.3%)
Histological Findings, n (%)	
Histologically Confirmed Autoimmune Gastritis (AIG)	21 (91.3%)
OLGA Stage I	8 (34.8%)
OLGA Stage II	8 (34.8%)
OLGA Stage III	4 (17.4%)
OLGA Stage IV	1 (4.3%)
Intestinal Metaplasia (Fundus/Body)	22 (95.7%)
H. pylori Status, n (%)	
H. pylori Positive	2 (8.7%)
H. pylori Negative	21 (91.3%)
Gastric Neoplastic Complications, n (%)	
Gastric Neuroendocrine Tumor (gNEN)	0 (0%)
Gastric Adenocarcinoma	0 (0%)
Autoimmune Comorbidities, n (%)	
Presence of Other Autoimmune Diseases	13 (56.5%)
- Autoimmune Thyroiditis	10 (43.5%)
- Rheumatological Diseases	4 (17.4%)
- Psoriasis	3 (13.0%)
- Inflammatory Bowel Disease	1 (4.3%)
- Celiac Disease	1 (4.3%)

In terms of liver disease diagnoses, PBC was the most prevalent condition, identified in 11/23 patients (47.8%). AIH was diagnosed in 6/23 (26.1%) of the APCA-positive group, while PSC was present in 2/23 patients (8.7%) of the cohort. No patients presented with an overlap syndrome of AIH and PBC or PSC-PBC. One patient (4.3%) was diagnosed with advanced undefined biliary cirrhosis.

**Figure 1:**
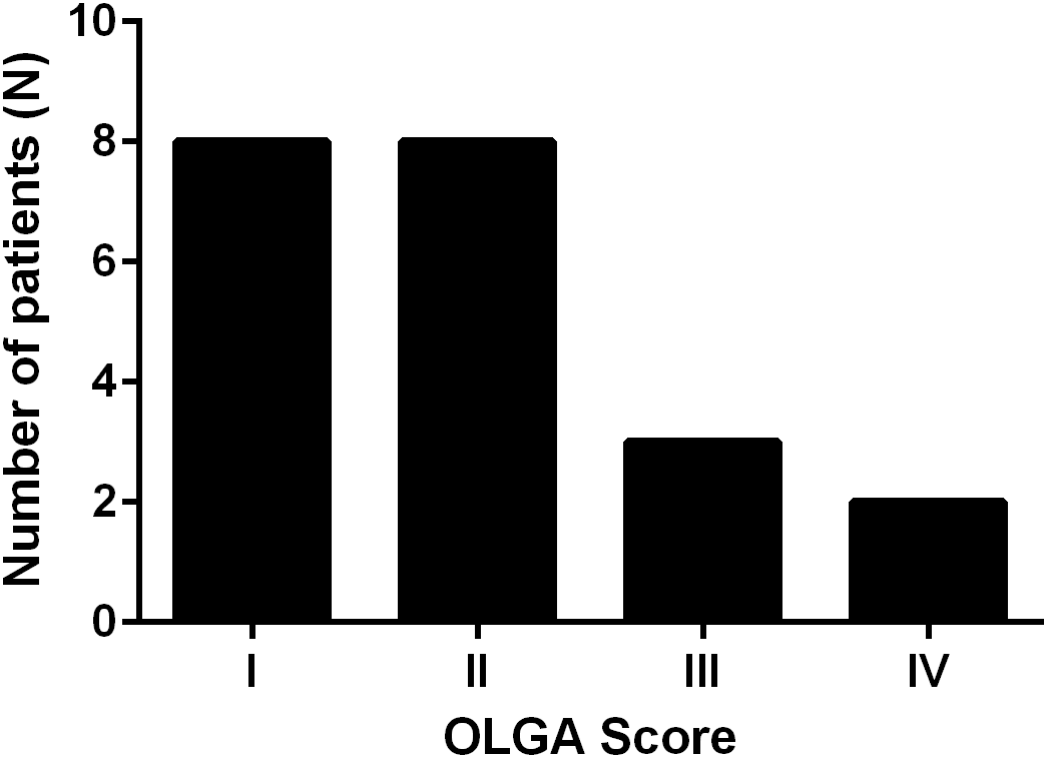
Distribution of OLGA (Operative Link on Gastritis Assessment) stages among APCA-positive patients. Most patients presented with early-stage atrophic gastritis (Stages I–II), while a smaller proportion exhibited advanced stages (III–IV).

In the APCA-positive cohort, 21 patients (91.3%) were confirmed to have histological atrophic gastritis, primarily affecting the body and fundus of the stomach. The severity of atrophic changes was assessed using the OLGA staging system, with the following distribution: Stage I: 8 patients; Stage II: 8 patients; Stage III: 4 patients; Stage IV: 1 patient (**Figure 1**).

Intestinal metaplasia was detected in 22 patients (95.7%) in the fundus/body and in none of the patients in the antrum. Two patients (8.7%) were found to be *H. pylori*-positive at the time of histological evaluation, while the remaining 21 patients (91.3%) were *H. pylori*-negative. Both *H. pylori-*positive patients underwent successful eradication therapy.

In the APCA-positive cohort, 13 patients (56.5%) exhibited comorbid autoimmune conditions.

The most commonly reported comorbidities were thyroid disease in 10 patients (43.5%), rheumatological diseases in four patients (17.4%), psoriasis in three (13.0%), inflammatory bowel disease in one (4.3%), and one patient (4.3%) had celiac disease.

When comparing the APCA-positive group to the overall AILD cohort, there were no statistically significant differences in age, gender, or autoimmune comorbidities. There was a slight difference, even if not statistically significant, in the distribution of AILD etiologies: PBC was more prevalent in the APCA-positive group (47.8%) compared to the overall AILD cohort (39.0%), while PSC was less prevalent in the APCA-positive group (8.7%) compared to the overall AILD cohort (13.3%) (**Table 3**).

**Table 3:** Comparison between overall AILD Cohort and APCA-positive Groups.

Characteristics	Overall AILD cohort (#pts= 105)	APCA-positive group (#pts= 23)	P-value
Age (median and range, years)	57 (20–88)	58 (20-77)	0.97
Gender (Female, %)	84 (80.0)	18 (78.3)	0.77
PBC (Number, %)	41 (39.0)	11 (47.8)	0.67
AIH (Number, %)	33 (31.4)	6 (26.1)	0.81
PSC (Number, %)	14 (13.3)	2 (8.7)	0.73
Overlap AIH-PBC (Number, %)	5 (4.8)	0 (0.0)	0.23
Autoimmune Comorbidities (Number, %)	52 (49.5)	13 (56.5)	0.84

### Gastric complications

Among the APCA-positive patients, no cases of gNENs or gastric adenocarcinoma were identified during the study period.

## Discussion

This study provides additional insights into the association between AILD and AIG, evaluated by the prevalence of APCA in patients with AILD. We found that 22.1% of the AILD cohort tested positive for APCA, indicating a noteworthy overlap between these two autoimmune conditions.

This aligns with preliminary findings reporting a similarly high prevalence of histologically confirmed AIG in AILD patients (32). Only other few case reports are published in Literature, showing the association between AIG and AILD (33–36).

Our findings support the hypothesis that AIG and AILD may share common autoimmune mechanisms and genetic predispositions, as previously suggested by other studies (33). The observed female predominance in APCA-positive patients (78.3%) is consistent with the broader epidemiological trend in autoimmune diseases (11). Also, the median age of APCA-positive patients was 58 years, aligning with the overall AILD cohort (57 years). Interestingly, the distribution of liver diseases within the APCA-positive group differed slightly from the overall AILD cohort. We noted indeed that PBC was relatively more frequent among APCA-positive patients compared to the overall AILD cohort. Only one preliminary cohort has reported similar trends, with PBC showing stronger associations with autoimmune gastritis compared to other liver autoimmune diseases (32). For context, the prevalence of PCA positivity in the general population is approximately 2–8%, increasing with age and more common in women (37). Moreover, in unselected PCA-positive individuals, the prevalence of histologically confirmed AIG is about 50–60%, with intestinal metaplasia being considerably less common than the 95.7% observed in our APCA-positive AILD cohort (38, 39). This suggests that AILD patients with PCA positivity may represent a higher-risk group for both AIG and intestinal metaplasia.

Notably, a significant proportion (56.5%) of AILD/APCA-positive patients exhibited other autoimmune comorbidities, including thyroid disease (43.5%), rheumatological diseases (17.4%), and psoriasis (13.0%). Thyroid disease, in particular, was the most common autoimmune comorbidity in this group (43.5%), consistent with previous studies showing that up to 40–50% of patients with AIG or atrophic gastritis have coexisting autoimmune thyroid diseases (40–42). Given this strong association, clinicians should consider routine screening for thyroid dysfunction in patients with AILD who test positive for APCA, independently from which type of AILD. This is supported by multiple studies showing a significantly increased prevalence of thyroid dysfunction among patients with AILD, especially PBC (43, 44). In line with this, a study by Liaskos et al. found that 31.8% of PBC patients had gastric parietal cell antibodies, a significantly higher prevalence than in other liver diseases, suggesting a distinct immunological profile for PBC in this context (45). Similarly, in the context of autoimmune gastritis, the strong bidirectional relationship with autoimmune thyroid disease—commonly referred to as “thyrogastric syndrome”—is well documented (46, 47). These associations reinforce the rationale for implementing targeted screening strategies in APCA-positive AILD patients, particularly those with PBC or concurrent thyroid autoimmunity.

In terms of gastric pathology, a high percentage of APCA-positive patients (91.3%) had histological evidence of atrophic gastritis affecting the body and fundus of the stomach. The OLGA staging system revealed varying degrees of severity, with most patients classified as Stage I or II, though a small number exhibited more advanced atrophy (Stage III and IV). Intestinal metaplasia was detected in 95.7% of patients, a finding that underscores the need for regular endoscopic surveillance (20) to monitor for neoplastic transformation. *H. pylori* status was assessed in all APCA-positive patients. Two patients (8.7%) were found to be *H. pylori*-positive at the time of histological assessment, while the remaining 21 patients (91.3%) were *H. pylori*-negative. In both *H. pylori*-positive patients, eradication therapy was successfully offered and completed. However, these patients exhibited corpus-restricted atrophic gastritis, showed oxyntic gland loss, and were APCA-positive, supporting the hypothesis that the *H. pylori* infection may have represented a superinfection on a background of AIG rather than being the primary driver of gastritis. This observation aligns with emerging evidence suggesting that *H. pylori* infection and autoimmune gastritis can occasionally coexist, particularly in the early phases of disease (48).

Importantly, none of the patients in this cohort were found to have gastric cancer or gNENs. This finding must be interpreted with caution due to the relatively small sample size and the cross-sectional design, which inherently limits the duration of clinical follow-up. Neoplastic transformation in AIG is known to occur over a prolonged period, and its absence at a single time point does not eliminate the possibility of future malignancy. This is supported by a long-term prospective study that found that 8.5% of patients with AIG developed neoplastic complications during the observation period (16, 49). Similarly, a meta-analysis by Chen et al. calculated an annual incidence of 0.14% for gastric cancer and 0.83% for type 1 gNENs in patients with autoimmune metaplastic atrophic gastritis, emphasizing the cumulative neoplastic risk over time (50). However, the association between AIG and gastric cancer remains controversial and is not yet fully established (51). In contrast, the link between AIG and gNENs is more consistently reported (19), although these tumors typically have a favorable prognosis. Therefore, the role of routine AIG screening in AILD patients as well as the decision to pursue gastric surveillance in this setting should be carefully individualized, and universal screening cannot currently be recommended. Further longitudinal studies with extended follow-up and larger AIG/AILD patient populations are needed to accurately assess the incidence of neoplastic risk in these patients.

The lack of significant statistical differences between the APCA-positive group and the overall AILD cohort in terms of age, gender, and autoimmune comorbidities suggests that the presence of APCA is not necessarily associated with more severe liver disease or a broader autoimmune profile. This is consistent with findings in autoimmune thyroid disease (ATD), where APCA positivity does not correlate strongly with gender or disease severity (52).

This study has several limitations that should be acknowledged. First, the relatively small sample size of the APCA-positive group (n=23) limits the statistical power and generalizability of the findings. Second, the study design introduces a potential work-up bias, as only APCA-positive patients underwent upper gastrointestinal endoscopy. Consequently, cases of AIG may have gone undetected among APCA-negative individuals, potentially underestimating the true prevalence of AIG in the broader AILD population. Similar limitations have been reported in prior studies assessing the overlap between autoimmune gastritis and liver autoimmunity, where selective screening restricted the accurate estimation of disease burden (16). To overcome these limitations, future research should probably implement a standardized endoscopic screening protocol across all AILD patients, irrespective of serologic status, to enable a more accurate characterization of AIG prevalence and its clinical implications.

Moreover, the cross-sectional design of this study limits the ability to establish temporal relationships between the onset of AILD, AIG, and associated autoimmune comorbidities. As a result, causality and disease progression patterns remain unclear. Longitudinal studies are necessary to determine whether APCA-positive patients face increased long-term risks, particularly concerning gastric neoplasms or the progression of liver pathology. Prior long-term data suggest that AIG-related complications, including gastric neuroendocrine tumors and atrophic progression, may take years to manifest, reinforcing the need for prospective follow-up (16).

In addition to observational data, future research should focus on elucidating the genetic and immunological mechanisms underpinning the overlap between AIG and AILD. Genome-wide association studies (GWAS) have already identified shared susceptibility loci across multiple autoimmune diseases, including variants in HLA regions, CTLA4, and IL2RA, suggesting common immunoregulatory pathways (27). Identifying disease-specific or shared biomarkers could facilitate risk stratification, earlier diagnosis, and tailored therapeutic approaches.

Finally, it remains to be determined whether immunomodulatory treatments commonly used in AILD—such as corticosteroids, azathioprine, or ursodeoxycholic acid—have a protective, neutral, or detrimental effect on the development or progression of AIG. Clarifying these therapeutic interactions could further support an integrated management strategy for patients with overlapping autoimmune phenotypes.

## Conclusion

In conclusion, this study highlights a significant overlap between AILDs and AIG, with a substantial proportion of AILD patients testing positive for APCA. The high prevalence of thyroid disease and other autoimmune comorbidities in APCA-positive patients underscores the need for a multidisciplinary approach to their management. Given the risks of gastric atrophy and intestinal metaplasia, cautious individualized evaluation for endoscopic surveillance may be considered in these patients, particularly in those with additional risk factors. Universal screening cannot currently be recommended based on our data and requires further confirmation in larger, longitudinal studies. Further research is needed to clarify the mechanisms linking AIG and AILD and to develop more targeted strategies for the diagnosis and management of these complex autoimmune conditions.

## Data Availability

The raw data supporting the conclusions of this article will be made available by the authors, without undue reservation.
